# 5′ tRNA halves are present as abundant complexes in serum, concentrated in blood cells, and modulated by aging and calorie restriction

**DOI:** 10.1186/1471-2164-14-298

**Published:** 2013-05-02

**Authors:** Joseph M Dhahbi, Stephen R Spindler, Hani Atamna, Amy Yamakawa, Dario Boffelli, Patricia Mote, David IK Martin

**Affiliations:** 1 Department of Biochemistry, University of California at Riverside, Riverside, CA, 92521, USA; 2 Department of Basic Sciences, Neuroscience, The Commonwealth Medical College, Scranton, Scranton, PA, 18510, USA; 3 Center for Genetics, Childrens Hospital Oakland Research Institute, Oakland, CA, 94609, USA

**Keywords:** Aging, Calorie restriction, Circulating small RNAs, tRNA derivatives, tRNA halves, Serum, Plasma

## Abstract

**Background:**

Small RNAs complex with proteins to mediate a variety of functions in animals and plants. Some small RNAs, particularly miRNAs, circulate in mammalian blood and may carry out a signaling function by entering target cells and modulating gene expression. The subject of this study is a set of circulating 30–33 nt RNAs that are processed derivatives of the 5′ ends of a small subset of tRNA genes, and closely resemble cellular tRNA derivatives (tRFs, tiRNAs, half-tRNAs, 5′ tRNA halves) previously shown to inhibit translation initiation in response to stress in cultured cells.

**Results:**

In sequencing small RNAs extracted from mouse serum, we identified abundant 5′ tRNA halves derived from a small subset of tRNAs, implying that they are produced by tRNA type-specific biogenesis and/or release. The 5′ tRNA halves are not in exosomes or microvesicles, but circulate as particles of 100–300 kDa. The size of these particles suggest that the 5′ tRNA halves are a component of a macromolecular complex; this is supported by the loss of 5′ tRNA halves from serum or plasma treated with EDTA, a chelating agent, but their retention in plasma anticoagulated with heparin or citrate. A survey of somatic tissues reveals that 5′ tRNA halves are concentrated within blood cells and hematopoietic tissues, but scant in other tissues, suggesting that they may be produced by blood cells. Serum levels of specific subtypes of 5′ tRNA halves change markedly with age, either up or down, and these changes can be prevented by calorie restriction.

**Conclusions:**

We demonstrate that 5′ tRNA halves circulate in the blood in a stable form, most likely as part of a nucleoprotein complex, and their serum levels are subject to regulation by age and calorie restriction. They may be produced by blood cells, but their cellular targets are not yet known. The characteristics of these circulating molecules, and their known function in suppression of translation initiation, suggest that they are a novel form of signaling molecule.

## Background

Several classes of small RNAs have been found to mediate biological functions in animals and plants [1-5]. miRNAs, siRNAs, piRNAs, and others are bound by Argonaute proteins, and have the common property of directing protein complexes to nucleic acids with sequence complementarity, where they may cleave or otherwise alter the target [6]. In both plants and animals, some small RNAs are able to travel between tissues within an organism, thus transferring their functions to other cells. In vertebrates, there has been much recent interest in the presence of specific miRNAs in the plasma and serum; there is some evidence that these can be taken up by cells and alter gene expression, and there is also interest in the possibility that they can be markers of specific disease states, including cancer [7-9].

There is also evidence for processing of non-coding RNAs into smaller RNAs, many with as yet poorly understood functions [10,11]. Many of the non-coding RNAs that appear to undergo processing into smaller RNAs have well studied functions, although their smaller derivatives often do not. In particular, tRNA is processed into shorter forms termed tRNA fragments (tRFs) [12,13]. The subject of this report is a tRNA fragment created by cleavage of tRNA near the anticodon loop to create a ″5′ tRNA half” (the term we will use here). Previous reports have described 5′ tRNA halves as intracellular molecules interacting with components of the translation initiation complex. 5′ tRNA halves have been shown to be induced by the ribonuclease angiogenin in response to stress in cultured cells, to promote assembly of stress granules carrying stalled preinitiation complexes, and to inhibit mRNA translation [14,15]; little more is known about their function.

We have sequenced small RNAs present in mouse serum; when multiple reportable alignments of the sequencing reads to the mouse genome were allowed, we noted the presence of a class of tRNA-derived 30–33 nt fragments that closely resemble the 5′ tRNA halves previously described in stressed cell cultures. Investigation of these 5′ tRNA halves reveals a novel class of circulating small RNAs whose characteristics, including changes with age that are antagonized by calorie restriction, strongly suggest physiologic regulation and function.

## Results and discussion

### Sequencing and computational analysis of small RNAs circulating in mouse serum

**Figure 1:**
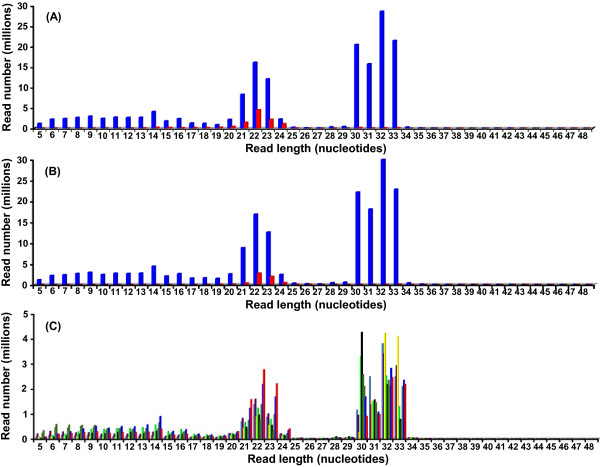
**Length distribution of reads obtained by deep sequencing of small RNAs extracted from mouse serum.** Shown here are only those reads that map to the mm10 (GRCm38) mouse genome. (**A**) Length distribution is displayed by abundance of sequencing reads combined from 9 serum samples. Reads were mapped to the mouse genome with bowtie according to Maq’s default policy, either allowing (blue bars) or disallowing (red bars) multiple reportable alignments for each read. (**B**) Combined reads were mapped to the mouse genome with bowtie according to the end-to-end k-difference policy, either allowing (blue bars) or disallowing (red bars) multiple reportable alignments for each read. (**C**) Length distribution of separate sequencing reads obtained from 9 individual serum small RNA samples. Length distribution is displayed by abundance of sequencing reads that were mapped to the mouse genome with bowtie according to Maq’s default policy, and allowing multiple reportable alignments for each read. Bars with different colors denote the source of the sequenced serum small RNA from the 9 different samples.

While investigating the effects of aging and calorie restriction (CR) on the profiles of cell-free small RNAs circulating in the bloodstream, we used small RNA-Seq (Illumina reads of 50 nt) to compare the serum levels of small RNAs from young and old control mice, and old mice subjected to CR. A combined total of 196,083,881 pre-processed sequencing reads obtained from 9 different serum samples, were mapped to the mouse genome with bowtie using parameters that align reads according to a policy similar to Maq’s default policy [16]. Alignment of the combined 196,083,881 pre-processed sequencing reads generated a dataset of 163,078,230 mapped reads (83.2%), ranging from 5 to 48 nt. The size distribution of the mapped reads revealed an expected peak at 20–24 nt consistent with the size of miRNAs (Figure 1).

Only if multiple reportable alignments are allowed during bowtie mapping does an unfamiliar second peak emerge at 30–33 nt (Figure 1A). The 30–33 nt peak persists when the bowtie alignment mode is changed from the Maq’s default policy (n option) to the end-to-end k-difference policy (v option), but again disappears when multiple reportable alignments are suppressed (Figure 1B). The same two-peak pattern was observed when the 9 individual sequenced serum small RNA samples were mapped to the mouse genome (Figure 1C). Dependence of the 30–33 nt peak on multiple reportable alignments indicates that the reads are encoded by repetitive DNA. Six percent of the 163,078,230 mapped reads, aligned to a group of RepeatMasker classes (DNA, LINE, LTR, Low complexity, RC, SINE, Satellite, and Simple repeat); these reads were mainly < 20 nt in size (Additional file 1: Figure S1) and were not considered for further analysis.

**Figure 2:**
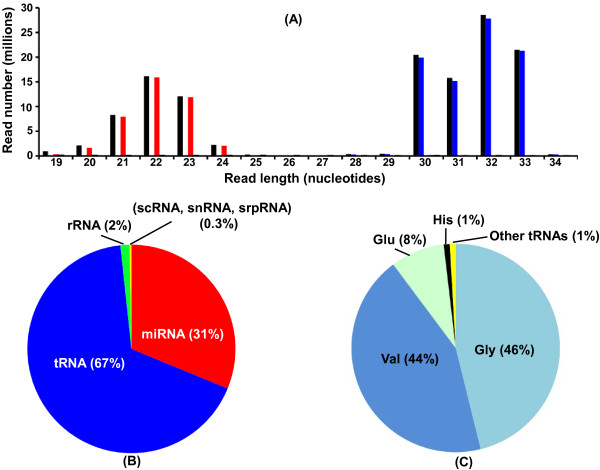
**Annotation of combined sequencing reads of small RNAs extracted from 9 mouse serum samples.** (**A**) Length distribution by abundance of the total reads mapped to the mouse genome (black bars), with reads annotated as mapping to either miRNAs (red bars), tRNAs (blue bars), rRNAs (green bars), or other small RNAs (yellow bars). Other small RNAs include scRNA, snRNA, and srpRNA. Note that the reads in the 20–24 nt and the 30–33 nt peaks are almost exclusively annotated as miRNAs and tRNAs, respectively. (**B**) A pie chart showing the percentage of reads mapping to the specific types of small RNAs. (**C**) Frequencies of 5′ tRNA half types represented in the aligned reads.

Annotation analysis of the mapped sequencing reads revealed that the 30–33 nt peak consists of reads mapping to tRNA genes (Figure 2A), which are present in multiple copies in the genome. Reads in the 20–24 nt peak were mostly annotated as miRNAs. Further analysis showed that of the total 163,078,230 reads that mapped to the mouse genome, 128,703,415 (79%) map to sequences encoding small RNAs, of which 67% and 31% were annotated as tRNAs and miRNAs, respectively (Figure 2B). The remaining < 3% of reads mapped to sequences annotated as encoding rRNA and other small RNAs (scRNA, snRNA, srpRNA).

### Characterization of circulating small RNAs derived from tRNAs

**Figure 3:**
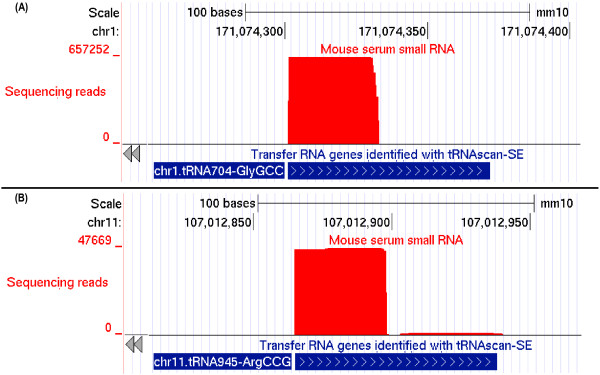
**UCSC genome browser screenshots illustrating alignment of reads to two tRNA genes.** Shown are the Illumina sequencing reads (red), and the tRNA genes (blue) as annotated in the tRNA genes track ”Transfer RNA genes identified with tRNAscan-SE“. (**A**) The alignment (number of reads, y-axis) shows that all the sequencing reads align to the 5′ end of chr1.tRNA704-GlyGCC gene. (**B**) The majority of the sequencing reads align to the 5′ end of chr11.tRNA945-ArgCCG gene, whereas only a very small number of sequencing reads aligns to the 3′ end.

Since the 86,343,437 reads that align to tRNA genes are only 30 to 33 nt, and thus do not represent full length tRNAs, we examined the tRNA end distribution of the reads, and annotated the reads based on their overlap with 5′ or 3′ ends of tRNAs. More than 99% of the tRNA-derived reads align with the 5′ end of a tRNA; this is exemplified in Figure 3 for two tRNA genes.

**Figure 4:**
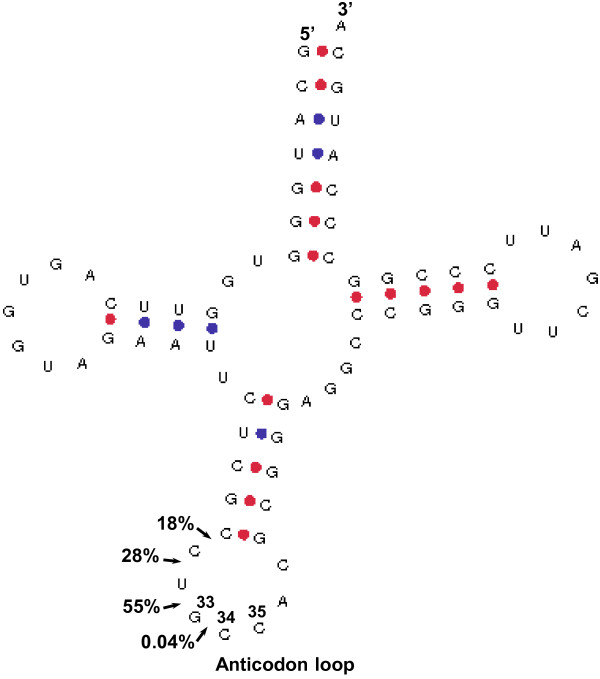
**Cleavage sites of tRNAs.** The cloverleaf structure of chr1.tRNA704-GlyGCC gene (downloaded from http://gtrnadb.ucsc.edu) showing cleavage sites at the anticodon loop, with the percentage of the reads that map to the 5′ end of the tRNA (arrowheads). The cleavage sites are upstream of the GCC anticodon located at nucleotides 33 to 35. Numbers inside the anticodon loop indicate anticodon nucleotides positions.

23%, 17%, 35%, and 26% of the sequencing reads that map to tRNAs are 30, 31, 32, and 33 nucleotide in size, respectively (Table 1), indicating that full length tRNAs are cleaved in the anticodon loop at more than one site and at varying rates to generate the 5′ tRNA halves found in serum. As an example, Figure 4 depicts the size frequency of reads mapping to the 5′ end of the chr1.tRNA704-GlyGCC gene; this indicates that this tRNA is cleaved at different rates and at 4 different sites located upstream of the GCC anticodon in the anticodon loop.

**Table 1:** Total number and percentage of the different sizes of sequencing reads that map to tRNAs

**Read size in nucleotides**	**Number of reads**	**Percentage of reads**
30	16649224	23%
31	12343893	17%
32	25190160	35%
33	18724475	26%

It is unlikely that this result is a sequencing artifact: the full length of most tRNAs is 75–90 nt, and the sequencing runs used to generate these data were 50 cycles while the reads occupy a narrow size range of 30–33 nt. This pattern suggests that the tRNA reads were derived from processed fragments of full length tRNAs; the remainder of the tRNA was not significantly detected in the serum small RNA libraries. In support of this conclusion, tRNAs have been shown to undergo cleavage within anticodon loops to produce tRNA-derived stress-induced fragments (tiRNAs) when cultured cells are subjected to stresses such as arsenite, heat shock, or ultraviolet irradiation [17,18]. Such cleavage of the anticodon loop does not seem to be part of a tRNA degradation process, because the generated 5′ tRNA fragments are stable in the cell. Our findings indicate that tRNA fragments highly similar to tiRNAs are present under normal (unstressed) conditions, and can remain stable even after they are released into the peripheral blood. 5′ but not 3′ tRNA fragments inhibit mRNA translation initiation in cultured cell lines [18].

The individual 5′ tRNA halves present in serum are derived from a small subset of tRNAs (Figure 2C). The most abundant circulating tRNA halves were derived from the isoacceptors of glycine (46%), valine (44%), glutamine (8%), and histidine (1%), and the remaining amino acids together represented <1%. We contrasted the number of tRNA genes in the Genomic tRNA Database [19], with the relative abundances of the circulating 5′ tRNA halves, and found no correlation (Table 2). For example, the most abundant circulating 5′ tRNA halves were derived from tRNA-Gly, and the copy number of tRNA-Gly gene is 29; on the other hand tRNA-Cys genes, with a copy number of 57, generate <1% of the 5′ tRNA-Cys halves.

**Table 2:** Frequencies of circulating 5′ tRNA halves and the gene copy number of tRNAs from which the tRNA halves were derived

**tRNA type**	**Gene copy number**	**% of circulating tRNA halves**
Gly	29	46%
Val	23	44%
Glu	21	8%
His	11	1%
Others*	351	< 1%

* All the remaining tRNAs combined.

This implies a tRNA type-specific biogenesis and/or release of the circulating 5′ tRNA halves.

### Presence in circulating mouse blood of particles containing stable cell-free 5′ tRNA halves

**Figure 5:**
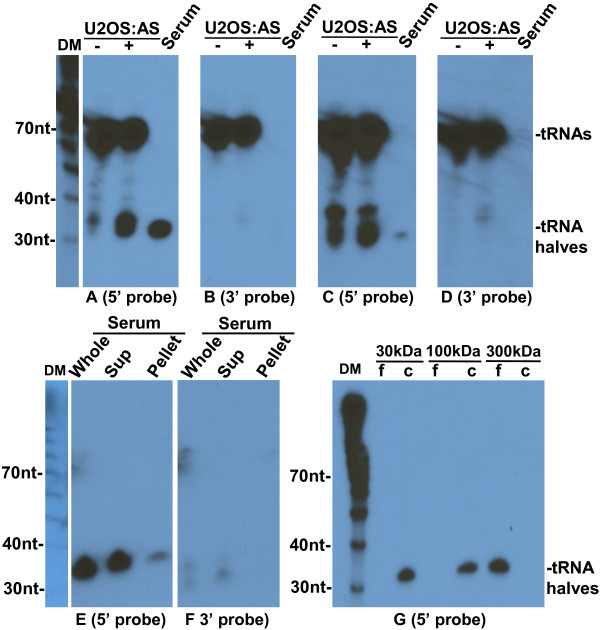
**Detection of 5′ tRNA halves in mouse serum.** Northern blot analysis of RNA extracted from U2OS cells cultured in the absence (−) or presence (+) of sodium arsenite (AS), or from 0.4 ml of mouse serum. The blot was hybridized to a ^32^P-end-labeled oligonucleotide probe complementary to the 5′ (**A**) or 3′ end (**B**) of tRNA-Gly-GCC. The blot was also hybridized to a ^32^P-end-labeled oligonucleotide probe complementary to the 5′ (**C**) or 3′ end (**D**) of tRNA-Val-CAC. 5′ tRNA halves were also detected in fractionated mouse serum. (**E**-**F**) Northern blotting analysis was carried out on RNA extracted from either 0.4 ml of mouse whole serum, from the supernatant (Sup) after ultracentrifugation of 0.4 ml of mouse serum at 110000g, or the ultracentrifugation pellet. The blot was hybridized to a ^32^P-end-labeled oligonucleotide probe complementary to the 5′ (**E**) or 3′ end (**F**) of tRNA-Gly-GCC. (**G**) Ultrafiltration indicates a size for tRNA serum particles between 100 and 300 kDa. Samples of 0.2 ml serum mixed with 1.8 ml PBS were subjected to ultrafiltration through Vivaspin 2 columns with 30, 100, and 300 kDa MW cut-offs. Total RNAs were extracted from filtrate (f) and concentrate (c) fractions. Blot was hybridized to ^32^P-end-labeled oligonucleotide probes complementary to the 5′ end of tRNA-Gly-GCC. The positions of full length tRNAs and tRNA halves are indicated on the right. DM: decade markers.

To obtain an independent validation of the sequencing results, we used Northern blotting to analyze small RNAs circulating in the mouse serum. As a positive control for detection of tRNA halves by Northern blotting, we included RNA from U2OS cells cultured in the absence or presence of sodium arsenite, which is known to generate tRNA halves in these cells [18]. We probed RNA from mouse serum with oligonucleotides complementary to 5′ or 3′ ends of specific tRNAs. Probes specific for the 5′ ends of tRNA-Gly-GCC or tRNA-Val-CAC detected a band migrating near the 30 nt RNA marker (Figure 5A and C) confirming the presence of stable circulating 5′ tRNA halves. No significant bands migrated with the 30 nt RNA marker when the same Northern blot was probed for the 3′ end of tRNA-Gly-GCC or tRNA-Val-CAC (Figure 5B and D).

We also probed RNA from mouse serum with a probe complementary to the 5′ end of tRNA-Asn-GTT to confirm the low abundance of circulating tRNA halves derived from tRNAs that were barely detected in the sequencing data. A 5-day exposure to X-ray film showed a very weak signal from tRNA-Asn-GTT probe compared to the strong signal from the tRNA-Gly-GCC probe obtained after a short (25 minute) exposure (Additional file 1: Figure S2). These results are consistent with the sequencing, and inconsistent with a sequencing bias. They imply a tRNA type-specific biogenesis and/or release of the circulating 5′ tRNA halves.

**Figure 6:**
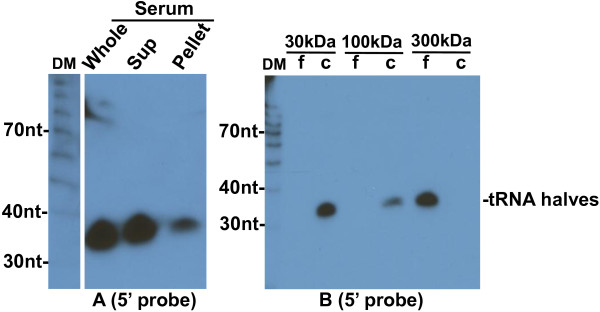
**Northern blotting analysis of tRNA-Val-CAC halves in mouse serum.** (**A**) RNA extracted from 0.4 ml of mouse whole serum, from supernatant or pellet after ultracentrifugation of 0.4 ml of mouse serum at 110000g and analyzed with northern blotting by hybridization to a ^32^P 5′-end labeled oligonucleotide probe complementary to the 5′ end of tRNA-Val-CAC. (**B**) RNA extracted from 0.2 ml serum mixed with 1.8 ml PBS subjected to ultrafiltration with Vivaspin 2 columns with 30, 100, and 300 kDa MW cut-off. Total RNAs were extracted from filtrate (f) and concentrate (c) fractions. Blot was hybridized to a ^32^P 5′-end labeled oligonucleotide probe complementary to the 5′ end of tRNA-Val-CAC. The positions of full length tRNAs and tRNA halves are indicated on the right. DM: decade markers.

We next asked if the 5′ tRNA halves are contained within circulating exosomes or microvesicles. We Northern blotted RNA extracted from pellet or supernatant after ultracentrifugation of mouse serum at 110000g for 2 hours. A probe for the 5′ end of tRNA-Gly-GCC detected an ~30 nt band present mainly in the supernatant and visible only as a trace in the pellet (Figure 5E), while a probe for the 3′ end did not detect any significant signal (Figure 5F). Identical results were obtained for the 5′ end of tRNA-Val-CAC (Figure 6A). These findings indicate that the 5′tRNA halves are mostly not included in exosomes or microvesicles, which would pellet in these conditions. Similarly, exosome encapsulation is not required for the stability of circulating miRNAs; after pelleting exosomes by ultracentrifugation of plasma, miRNAs were still detected in the supernatant fraction [20,21].

Because the tRNA halves we observe are stable in circulation but not encapsulated in exosomes, they are most likely complexed to carrying factors (e.g., proteins that protect them from degradation). To determine the size range of the putative complexes carrying the 5′ tRNA halves in the serum, we Northern blotted RNA extracted from concentrate or filtrate fractions after ultrafiltration of mouse serum samples through Vivaspin 2 columns with 30, 100, or 300 kDa MW cut-off. A probe for the 5′ end of tRNA-Gly-GCC detected a ~30 nt band in the concentrates of 30 and 100 kDa MW cut-off, and in the filtrate of 300 kDa MW cut-off (Figure 5G). Identical results were obtained for the 5′ end of tRNA-Val-CAC (Figure 6B).

Thus 5′ tRNA halves circulate as part of 100–300 kDa complexes, while the 5′ tRNA halves themselves are only ~10 kDa. This is reminiscent of reports that miRNAs can circulate in the bloodstream as components of RNA-protein/lipoprotein complexes. Stable argonaute2-miRNA complexes that are not part of microvesicles were recovered from plasma and serum, and high-density lipoprotein has been reported to carry and deliver miRNAs to recipient cells [20-22].

### 5′ tRNA halves are concentrated in hematopoietic and lymphoid tissues

**Figure 7:**
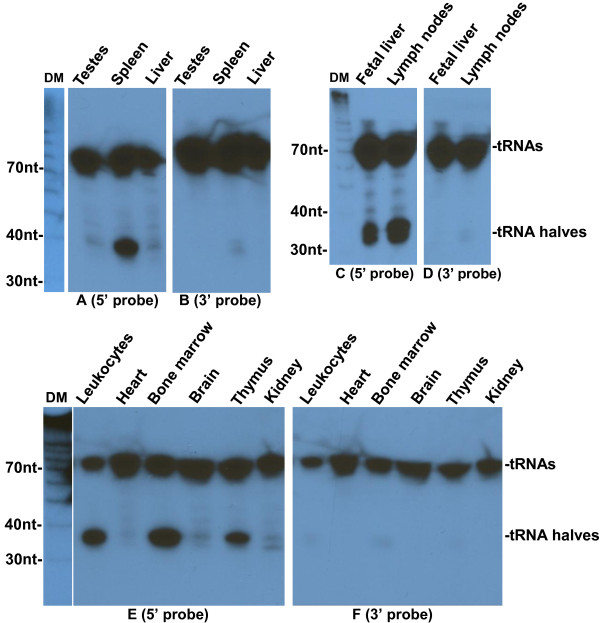
**Tissue distribution of tRNA-Gly-GCC halves.** Northern blotting analysis of RNA extracted from the indicated mouse tissues. Blots were hybridized with ^32^P-end-labeled oligonucleotide probes complementary to the 5′ (**A, C**, and **E**) or 3′ end (**B, D**, and **F**) of tRNA-Gly-GCC. The positions of full length tRNAs and tRNA halves are indicated on the right. DM: decade markers.

**Figure 8:**
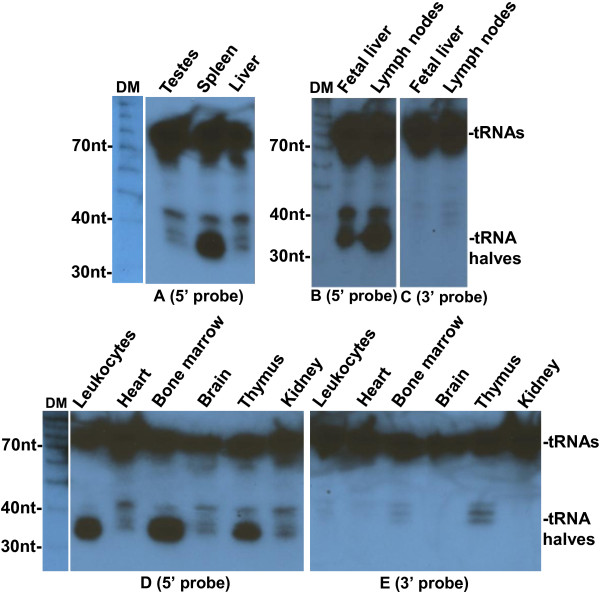
**Tissue distribution of tRNA-Val-CAC halves.** Northern blotting analysis of RNA extracted from the indicated mouse tissues. Blots were hybridized with ^32^P-end-labeled oligonucleotide probes complementary to the 5′ (**A**, **B**, and **D**) or 3′ end (**C** and **E**) of tRNA-Val-CAC. The positions of full length tRNAs and tRNA halves are indicated on the right. DM: decade markers.

To investigate whether 5′ tRNA halves are present in tissues we extracted total RNA from liver, spleen, and testes, and did Northern blots with probes complementary to 5′ and 3′ ends of tRNAs. We detected tRNA halves with a probe complementary to the 5′ end of tRNA-Gly-GCC in the spleen, but not in the liver and testes; a probe for the 3′ end tRNAs detected only full length tRNAs in all 3 tissues (Figure 7A-B). This prompted us to explore the possibility that 5′ tRNA halves are present specifically in hematopoietic tissues. Northern blotting of several mouse tissues confirmed that 5′ tRNA halves are present in hematopoietic and lymphoid tissues including spleen, lymph nodes, fetal liver, leukocytes, bone marrow, and thymus, but almost absent in non-immune tissues including testes, liver, heart, brain, and kidney (Figure 7); the presence of 3′ tRNA halves was not significant in any tissue. This finding is consistent with a previous report [23], in which tRNA halves were unexpectedly detected on cloning of microRNAs from human fetal liver, which is the main hematopoietic organ during fetal development. Identical results were obtained when the same Northern blots were probed for the 5′ and 3′ ends of tRNA-Val-CAC (Figure 8).

More extensive studies will establish if 5′ tRNA halves are concentrated in particular blood cell types, although the very high levels in lymph nodes point to lymphocytes as one such type. The evidence does not establish whether the 5′ tRNA halves are concentrated in hematopoietic cells because they are produced there, or because they are preferentially taken up from the blood: neither the origin nor the destinations of the 5′ tRNA halves is certain. The low levels of 5′ tRNA halves present in non-hematopoietic tissues may indicate low levels in those tissues, but they may also be derived from residual blood cells in those tissues.

### A chelating agent destabilizes circulating 5′ tRNA halves

**Figure 9:**
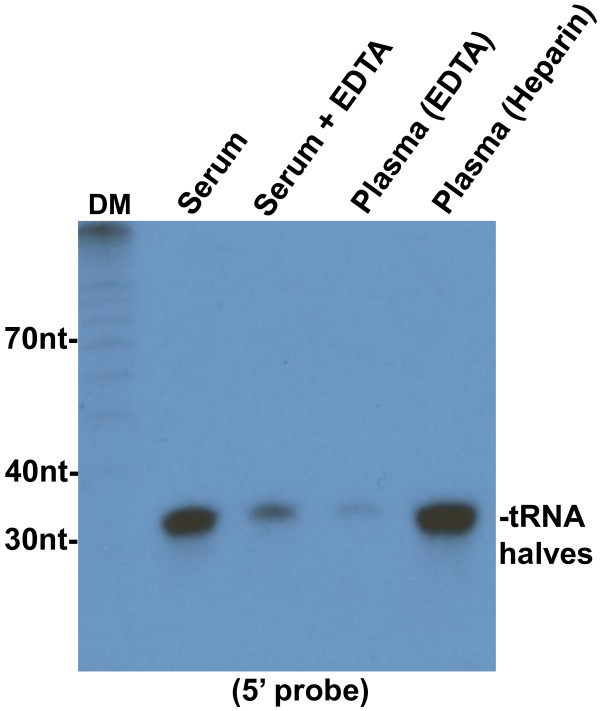
**Detection of 5′ tRNA halves in mouse plasma and serum.** Northern blot of RNAs extracted from 0.4 ml of mouse serum, serum treated with EDTA, and heparin- or EDTA-collected plasma, hybridized to a ^32^P 5′-end labeled oligonucleotide probe complementary to the 5′ end of tRNA-Gly-GCC. EDTA sharply lowers the abundance of 5′ tRNA halves in serum; in plasma, 5′ tRNA halves are abundant when heparin is the anticoagulant, but are nearly absent when EDTA is present. 5′ tRNA halves are similarly abundant when calcium citrate is used as the anticoagulant (not shown).

**Figure 10:**
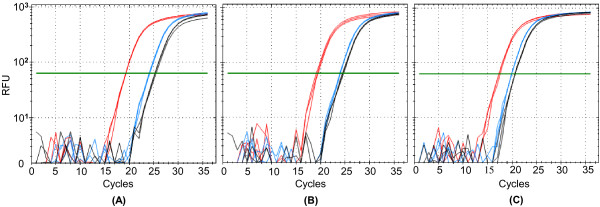
**Real-time PCR amplification plots of circulating miRNAs.** Shown are the amplification plots for miR-16 (blue), miR-24 (black), and the spiked-in miR-Cel-39 (red) measured in mouse serum (**A**), mouse serum treated with EDTA (**B**), and mouse plasma collected on EDTA (**C**). The y-axis represents the relative fluorescence units (RFU) in a semi-log scale. The x-axis represents the cycle at which fluorescence was detected above an automatically determined threshold for the indicated miRNA. EDTA does not change the concentration of miRNAs in plasma.

Because clotting has the potential to release particles that are not present in circulating blood, we asked if 5′ tRNA halves circulating in the mouse serum are also present in mouse plasma. Northern blotting with a 5′ tRNA half probe gave a very weak band in a plasma sample when compared to the band derived from an equal volume of serum from the same mouse (Figure 9). The lack of 5′ tRNA halves in plasma is not due to a global loss of small RNAs during preparation of the plasma, which was anticoagulated with EDTA. We used qPCR to assess the integrity of two circulating miRNAs in mouse serum, serum treated with EDTA, and plasma collected with EDTA. As shown by amplification in all three specimens (Figure 10), EDTA does not affect these circulating miRNAs.

**Figure 11:**
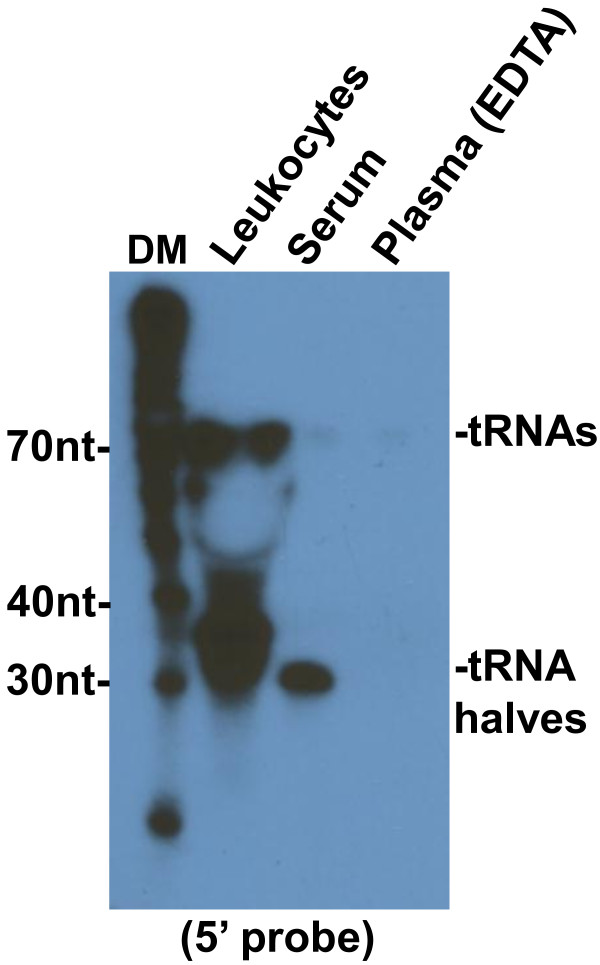
**5′ tRNA halves are also present in human serum and leukocytes, but not in EDTA plasma.** RNAs extracted from human leukocytes, 0.4 ml of human serum, or EDTA-collected plasma. The northern blot was hybridized to a ^32^P 5′-end labeled oligonucleotide probe complementary to the 5′ end of tRNA-Gly-GCC. The positions of full length tRNAs and tRNA halves are indicated on the right. DM: decade markers.

This result could suggest that 5′ tRNA halves are an artifact of blood clotting, but could also be an effect of EDTA, a chelating agent that depletes ions required for clotting. To assess the effects of EDTA on 5′ tRNA halves, we used Northern blotting to analyse a sample of serum that was incubated with EDTA for 15 min before RNA extraction. We also analyzed a sample of plasma extracted from blood collected with heparin, a nonchelating anticoagulant. This analysis showed that treatment of serum with EDTA significantly decreased the signal corresponding the 5′ tRNA halves, while 5′ tRNA halves are abundant in heparinized plasma (Figure 9). The same results were obtained with RNAs from human plasma collected on EDTA and from serum (Figure 11). These findings suggest that chelation of ions by EDTA destabilizes the complexes carrying 5′ tRNA halves, exposing the RNA to ribonucleases which are abundant in plasma.

### Calorie restriction offsets age-associated changes in levels of specific circulating 5′ tRNA halves

Calorie restriction (CR) can delay, prevent, or reverse many age-associated changes in physiologic parameters. We used aging and CR as model physiologic states to explore the possibility that they are associated with changes in the levels of circulating 5′ tRNA halves. We performed pairwise comparisons between young and old control groups to measure the differential abundance in circulating 5′ tRNA halves associated with old age, and between old control and old CR groups to determine whether CR has an effect on any age-associated changes.

This analysis revealed that aging is associated with alterations, either increase or decrease, in the circulating levels of 5′ tRNA halves derived from specific tRNA isoacceptors (Table 3). Notably, CR mitigated most of these age-related changes (Table 3), although it did not completely prevent them. CR has been shown to oppose the molecular and biological markers of aging including alterations in gene expression [24]. A causal relationship between circulating 5′ tRNA halves and the manifestations of aging is not established by this study, but it does indicate that levels are regulated in an age-associated fashion.

**Table 3:** Age-associated changes in the levels of mouse circulating 5′ tRNA halves and the effects of CR on the age-associated changes

				**Age**		**CR**	
tRNA^*^	**Young control **^**†**^	**Old control**^**†**^	**Old CR**^**†**^	**FC**^**‡**^	**p-value**	**FC**^**‡**^	**p-value**
**His-GTG**							
chr4:82619623-82619694	797	2988	1554	3.8	3.1E-11	−2	6.8E-04
chr2:122377363-122377434	798	2994	1549	3.8	3.7E-11	−2	6.4E-04
chr3:96452495-96452566	307	1140	590	3.8	3.8E-11	−2	6.1E-04
chr2:122375494-122375565	808	2990	1533	3.8	3.9E-11	−2	5.0E-04
chr2:122377968-122378039	309	1163	600	3.8	4.2E-11	−2	6.6E-04
chr3:96458070-96458141	802	2993	1523	3.8	4.3E-11	−2	4.8E-04
chr3:96500366-96500437	796	2954	1524	3.8	4.5E-11	−2	5.9E-04
chr3:96410069-96410140	301	1148	590	3.9	2.0E-11	−2	5.5E-04
**Arg-CCG**							
chr11:107012866-107012938	1243	256	302	−5	9.9E-12	1.2	4.7E-01
**Cys-GCA**							
chr11:97798906-97798977	933	370	688	−2.6	2.5E-06	1.8	2.4E-03
chr11:97988246-97988317	928	376	700	−2.5	3.9E-06	1.8	2.2E-03
chr11:97988923-97988994	930	360	684	−2.6	1.3E-06	1.9	1.5E-03
**Gly-GCC**							
chr1:171074302-171074372	16868	3739	3807	−4.5	3.5E-14	−1	9.6E-01
chr1:171066631-171066701	16820	3730	3790	−4.5	4.3E-14	−1	9.6E-01
chr1:171081876-171081946	16779	3725	3788	−4.4	5.3E-14	−1	9.6E-01
**Lys-CTT**							
chr17:23533962-23534034	4175	1939	3286	−2.2	8.8E-06	1.7	3.7E-03
chr3:96428235-96428307	4215	1964	3353	−2.2	1.0E-05	1.7	3.3E-03
chr17:23547360-23547432	4098	1923	3272	−2.2	1.1E-05	1.7	3.4E-03
chr17:23535332-23535404	14085	6569	11132	−2.2	1.2E-05	1.7	4.1E-03
chr13:23436340-23436412	4181	1962	3321	−2.2	1.2E-05	1.7	3.9E-03
chr3:96499512-96499584	13865	6507	11017	−2.2	1.3E-05	1.7	3.9E-03
chr11:48833883-48833955	13905	6539	11051	−2.2	1.4E-05	1.7	4.1E-03
**Val-AAC**							
chr13:23401073-23401145	1247	451	814	−2.8	3.3E-07	1.8	4.1E-03
chr13:23413248-23413320	1246	467	836	−2.7	5.4E-07	1.8	4.0E-03

^*^tRNA isoacceptor identity with corresponding genomic positions of the tRNA genes in the mouse mm10 genome.

^**†**^Average tRNA read count for the indicated experimental group reported as counts per million (cpm) reads in the sequenced library from the indicated experimental group.

^**‡**^Fold change calculated by EdgeR from pairwise comparisons between the young and old control groups for the age effect, or between the old control and old CR groups for the CR effect.

## Conclusions

Deep sequencing of small RNAs extracted from mouse serum identifies a population of tRNA-derived molecules, termed 5′ tRNA halves, previously described only as stress-induced inhibitors of translation initiation in cultured cells. 5′ tRNA halves are more abundant than miRNAs in mouse serum, and are derived from distinct subset of tRNAs by cleavage near the anticodon loop; the 3′ portion of the tRNA molecule is present in serum only in trace quantities. Ultracentrifugation and size fractionation establish that the 5′ tRNA halves circulate as part of a larger complex, but are not contained in exosomes or microvesicles; their sensitivity to the chelating agent EDTA provides further evidence that they exist as circulating nucleoprotein complexes. They are concentrated in hematopoietic and lymphoid tissues, and present in other tissues at very low levels that may reflect residual blood cells. The origin of the serum particles, and their destinations, are uncertain; however their concentration in blood cells suggest that they may be produced by these cells. Levels of serum 5′ tRNA halves are distinctly changed in aged mice, and calorie restriction inhibits these changes, indicating that they are subject to physiologic regulation. Taken together with the extant evidence that 5′ tRNA halves can regulate mRNA translation, the characteristics of the circulating 5′ tRNA halves we have discovered suggest that they function as signaling molecules with as yet unknown physiologic roles.

To date, the only known function of 5′ tRNA halves is inhibition of translation in cultured cells subjected to a variety of stressors; transfection of 5′ tRNA halves inhibits global translation in U2OS cells [14,18]. A study published while this paper was in preparation reported induction of 5′ tRNA halves in human airway epithelial cells upon infection with respiratory syncytial virus (RSV). Induction involves cleavage at the tRNA anticodon loop by angiogenin, and at least one type, the 5′ tRNA-Glu-CTC half, promotes RSV replication [25]. Our findings indicate that 5′ tRNA halves function on an organismal rather than merely a cellular level. Furthermore they are likely to function in a context much broader than cellular stress or infection: we find 5′ tRNA halves in unstressed conditions. Changes in their expression (either increased or decreased) with age are also consistent with a broader physiologic role, and it is particularly interesting that these changes are partially mitigated by calorie restriction.

The most extensively studied cellular tRNA halves are generated under stress conditions by angiogenin, which cleaves mature tRNAs within the anticodon loops [26]. The stress-induced tRNA halves target the translation initiation machinery to reprogram protein translation in order to promote cell survival during stress [14,26]. Pull-down and mass spectrometry analyses of RNA-protein complexes have identified several cellular proteins (YB-1, FXR-1, and PABP1) bound to intracellular 5′ tRNA halves [14]. The nature of the proteins and/or other factors that bind and stabilize the extracellular form of 5′ tRNAs halves has yet to be elucidated. Understanding of the origin, composition, and destinations of these complexes will provide insights into their role in organismal physiology.

## Methods

### Serum collection, RNA isolation, and small RNA library construction

Male mice of the long-lived B6C3F1 strain were fed either control or calorie-restricted (CR) diet (∼40% fewer calories than the control). Three mice were studied from each of three groups: young (7-month) and old (27-month) mice fed the control diet, and old (27-month) mice fed the CR diet. Total RNA including small RNA was isolated from each serum sample with miRNeasy kit (Qiagen) and used to construct indexed sequencing libraries with the Illumina TruSeq Small RNA Sample Prep Kit. The libraries were pooled and sequenced on an Illumina HiSeq 2000 instrument to generate 50 base reads. Further details about the mice and diets, and library construction are provided in SI Methods and Figures.

### Mapping and annotation of sequencing reads

Sequencing reads were pre-processed with FASTX-Toolkit (hannonlab.cshl.edu) to trim the adaptor sequences, and discard low quality reads. The obtained clean reads were mapped to the mouse reference genome (GRCm38/mm10) with bowtie version 0.12.8 [16] using different combinations of alignment and reporting options. We used the option ”-n 0 -l 14“ to align the sequencing reads according to a policy similar to Maq‘s default policy and requiring no mismatches in the first 14 bases (the high-quality end of the read). In addition, this mode of alignment was combined with options that define which and how many alignments should be reported; the option “-k 1 --best” instructed bowtie to report only the best alignment if more than one valid alignment exists, while the option “-m 1” instructed bowtie to refrain from reporting any alignments for reads having multiple reportable alignments. The “-k 1 --best” and “-m 1” modes of alignment reporting were also used in combination with the end-to-end k-difference (−v) alignment mode. Varying the alignment and reporting modes allowed the differential detection of two predominant peak sizes of sequencing reads as described in the results section.

Annotation analysis of the mapped sequencing reads was performed with bedtools [27] using the following databases: the Genomic tRNA Database [19] (gtrnadb.ucsc.edu), miRBase 18 (mirbase.org), and rRNA, snRNA, scRNA, and srpRNA which were extracted from the RepeatMasker track (genome.ucsc.edu; mm10).

### Analysis of differentially abundant circulating tRNA halves

The bowtie alignment files generated above from the young and old control and old CR serum sequencing samples were analyzed with bedtools [27] to obtain the coverage of the tRNA genes included in the Genomic tRNA Database [19] (gtrnadb.ucsc.edu), and to determine the read count for each tRNA in the database. The tRNA read counts were further analyzed with the Bioconductor package edgeR [28] to detect the changes in the levels of circulating 5′ tRNA halves in the different experimental groups. The algorithm of edgeR fits a negative binomial model to the count data, estimates dispersion, and measures differences using the generalized linear model likelihood ratio test which is recommended for experiments with multiple factors, such as the simultaneous analysis of age and diet in our study. The fitted count data was analyzed by performing pairwise comparisons between the different experimental groups: young and old control groups were compared to measure the differential abundance in circulating 5′ tRNA halves associated with old age; old control and old CR groups were compared to determine whether CR has an effect on any age-associated changes. The results were further filtered to keep only 5′ tRNA halves that achieved a minimum of 500 counts per million (cpm) in at least one of the 3 experimental groups.

### Northern blot assays

RNAs analyzed with Northern blots were extracted from normal or sodium arsenite-treated U2OS and from a variety of tissues and sera harvested from one-year-old mice fed control diet. Before RNA extraction, some serum samples were centrifuged at 110000 g for 2 hrs, and supernatant and pellet fractions were separated, or were separated into concentrate and filtrate fractions by ultrafiltration through Vivaspin 2 columns with 30, 100, or 300 kDa MW cut-off. RNAs were separated on 15% denaturing polyacrylamide gels, transferred and fixed to a membrane by chemical cross-linking [29], and hybridized with probes complementary to 5’ and 3′ ends of tRNAs. Further details about Northern blot assays, and probe sequences are provided in SI Methods and Figures.

## Competing interests

The authors declare that they have no competing interests.

## Authors’ contributions

J.M.D conceived and designed the study, carried out the bioinformatics analysis, and drafted the manuscript. SRS participated in the design of the study and the interpretation of the results. HA participated in the data analysis and interpretation of the results. AY, DB, and PM performed the experiments. DIKM participated in the interpretation of the results, and helped to draft the manuscript. All authors read and approved the final manuscript.

## Supplementary Material

Additional file 1 Additional Methods and Figures S1 and S2. Click here for file
