# Supplementary material for: 5′ tRNA halves are present as abundant complexes in serum, concentrated in blood cells, and modulated by aging and calorie restriction
Source: BMC Genomics. 2013 May 2;14:298. doi: 10.1186/1471-2164-14-298 (PMC3654920; doi:10.1186/1471-2164-14-298)
Supplement: Additional file 1 — Additional Methods and Figures S1 and S2. [file 1471-2164-14-298-S1.pdf]

MATERIALS AND METHODS

Mice and diets. One-month-old male mice of the long-lived B6C3F1 strain were purchased from Harlan (Indianapolis, IN). One week after arrival, mice were individually housed and randomly assigned to one of two groups, control or calorie restricted (CR). Control mice were fed 93 kcal/wk of a defined control diet (AIN-93M, diet no. F05312, BIO-SERV). CR mice were fed 52.2 kcal/wk of a defined CR diet (AIN-93M 40% Restricted, diet no. F05314, BIO-SERV). The CR mice consumed ~40% fewer calories than the control group. The CR diet was enriched so that the CR mice consumed approximately the same amount of protein, vitamins, and minerals per gram of body weight as the control mice. All mice had free access to water. Mice were maintained at 20–24°C and 50–60% humidity with lights on from 0600 to 1800 h. Sentinel mice were kept in the same room as the experimental mice, and serum samples were screened every 6 months for titers against 11 common pathogens. No positive titers were found during these studies. At 27-months of age, mice were euthanized, and blood was collected through cardiac puncture and processed immediately. A group of control mice were euthanized at 7 months of age and used as a young control group. Each group consisted of 3 mice. The Institutional Animal Care and Use Committee of the University of California, Riverside, approved animal protocols.

Serum collection, RNA isolation, and small RNA library construction. Immediately after collection, blood was transferred to BD Microtainer tubes (Becton, Dickinson and Company), incubated for 30 min at room temperature to allow blood clotting, and centrifuged at 5,000 g for 10 min. The serum supernatant was transferred to new tubes, centrifuged at 16,000 g for 15 min to remove any residual cells and cell-debris, and stored at -80 °C before use. Isolation of total RNA including small RNA was performed with miRNeasy kit (Qiagen) according to the manufacturer’s protocol with the exceptions of mixing 2 mL of Qiazol reagent with 0.4 mL serum, loading the entire aqueous phase onto a single column from the MinElute Cleanup Kit (Qiagen), and eluting the RNA in 20 µL of RNase-free water.

One fourth (5 µL) of the RNA isolated from each serum sample was used to construct sequencing libraries with the Illumina TruSeq Small RNA Sample Prep Kit, following the manufacturer’s protocol. Briefly, 3′ and 5′ adapters were sequentially ligated to small RNA molecules and the obtained ligation products were subjected to a reverse transcription reaction to create single stranded cDNA. To selectively enrich those fragments that have adapter molecules on both ends, the cDNA was amplified with 15 PCR cycles using a common primer and a primer containing an index tag; this allows multiplexing and sequencing different samples in a single lane of a flowcell. The amplified cDNA constructs were gel purified, and validated by checking the size, purity, and concentration of the amplicons on the Agilent Bioanalyzer High Sensitivity DNA chip. The libraries were pooled in equimolar amounts, and sequenced on an Illumina HiSeq 2000 instrument to generate 50 base reads. Image deconvolution and quality values calculation were performed using the modules of the Illumina pipeline.

RNA extraction from mouse tissues, stressed U2OS cells, fractionated mouse serum and plasma for Northern blot analysis. For stress induction, U2OS cells were cultured in McCoy's 5A Medium supplemented with 10% fetal calf serum and 1% of penicillin/streptomycin, and treated with 500 µM of sodium arsenite (Sigma) for 2 hours before RNA extraction. Tissues and sera were collected from one-year-old mice fed

control diet. Tissues were flash frozen in liquid nitrogen. Serum samples were centrifuged at 110,000 g for 2 hrs, and supernatant and pellet fractions were separated. Samples of 0.2 ml serum mixed with 1.8 ml PBS were subjected to ultrafiltration through Vivaspin 2 columns (GE Healthcare) with 30, 100, or 300 kDa MW cut-off, and concentrate and filtrate fractions were collected. All samples were stored at -80 °C before RNA extraction. For plasma preparation, mouse blood samples were mixed with 0.5M EDTA (10 µl/ml) or sodium heparin (5.5 mg/ml) and centrifuged at 10,000 g for 10 min. The plasma supernatant was transferred to new tubes, centrifuged at 16,000 g for 15 min to remove any residual cells and cell-debris, and stored at -80 °C before use. Total RNA including small RNA was isolated from tissue samples, cell pellets or serum fractions with miRNeasy kit (Qiagen).

Collection of human blood and RNA extraction from serum and plasma. Human blood samples were collected with Institutional Review Board approval after obtaining informed consent. Blood was collected from one young adult male in BD Vacutainer Venous Blood Collection Tubes (BD Diagnostics): K2 EDTA Spray-Dried (BD-366643) or Spray-Coated Sodium Heparin (BD367874). Blood was transferred to Leucosep Centrifuge Tubes (Grenier Bio One #227290P) and centrifuged at 800 g for 15 min at room temperature. The plasma supernatant was transferred to fresh tubes, centrifuged at 16,000 g for 15 min to remove any residual cells and cell-debris, and stored at -80 °C before use. Total RNA including small RNA was isolated from plasma or serum with miRNeasy kit (Qiagen).

Preparation of leukocytes from mouse and human blood and RNA extraction.

Blood was collected on EDTA, centrifuged at 1000 g for 15 minutes to separate the plasma and blood cells. The buffy coat was collected, incubated in erythrocyte lysis buffer (Qiagen), and washed with PBS. Leukocyte pellets were flash frozen in liquid nitrogen, and stored at -80 °C before use. Total RNA including small RNA was isolated from leukocytes pellets with miRNeasy kit (Qiagen).

Northern blot assays. RNAs extracted from tissue samples, cell pellets or serum fractions as described above were separated on 15 % polyacrylamide Criterion TBE-Urea gels (Bio-Rad), transferred to a Hybond NX membrane (GE life sciences), and fixed to the membrane by chemical cross-linking (1). Blots were hybridized overnight at 42 °C in ULTRAhyb-Oligo Buffer (Invitrogen) with the following ^{32}P-5'-end labeled oligonucleotide probes against the 5' end of tRNA-Gly-GCC (5'-GGCGAGAATTCTACCACTGAACCACCAA), the 3' end of tRNA-Gly-GCC (5'-TGCATTGGCCGGGAACCGAACCCGGGCCTCCCGCG), the 5' end of tRNA-Val-CAC (5'-AGGCGAACGTGATAACCACTACACTACGGA), or the 3' end of tRNA-Val-CAC (5'-TGTTTCCGCCCGGTTTCGAACCGGGGACCTTTCGCG), or the 5' end of tRNA- Asn-GTT (5'-CGAACGCGCTAACCGATTGCGCCACAGA). Membranes were washed twice with 2X SSC, 0.1 % SDS solution for 30 minutes, and exposed to X-ray films for detection of signals.

Real time quantitative PCR (qPCR). For qPCR assays, 10 fmoles of the synthetic C. elegans cel-miR-39 (Qiagen #MSY0000010) were spiked into 0.2 ml of serum or plasma before RNA extraction to account for variations during RNA extraction, cDNA synthesis, and real-time PCR. One fourth of total RNA extracted from 0.2 ml serum or plasma was reverse transcribed using the miScript Reverse Transcription Kit (Qiagen) according to the manufacturer's protocol. The obtained reverse transcription product was

amplified using the following Qiagen reagents: SYBR Green PCR Master Mix, Universal Primer, and miScript Primer Assays for miR-16, miR-24, and miR-Cel-39. Real-time qPCR was carried out on a Bio-Rad CFX96 thermocycler.

1. Pall GS & Hamilton AJ (2008) Improved northern blot method for enhanced detection of small RNA. *Nature protocols* 3(6):1077-1084.

SI FIGURES

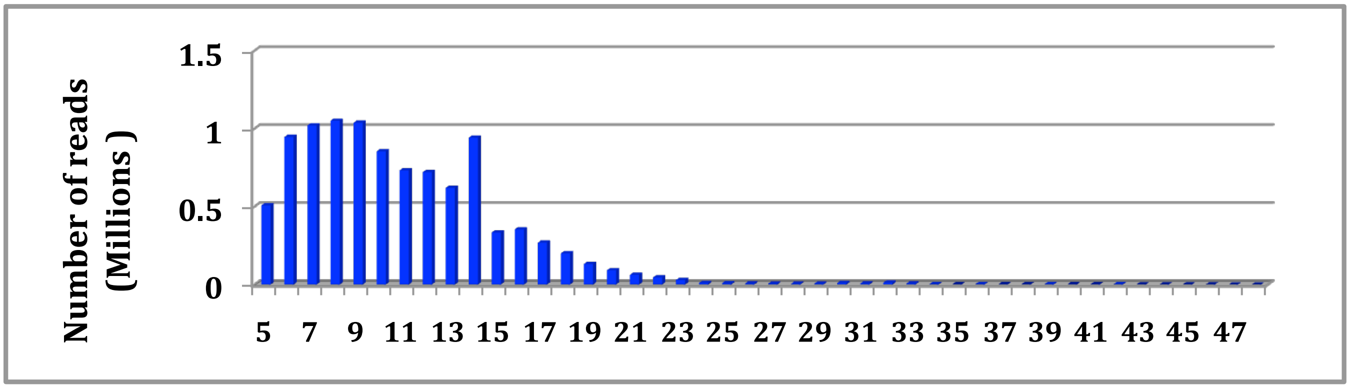

Figure S1 - Length distribution of sequencing reads that mapped to the RepeatMasker classes of DNA, LINE, LTR, Low_complexity, RC, SINE, Satellite, and Simple_repeat. Read length distribution is displayed by abundance of sequencing.

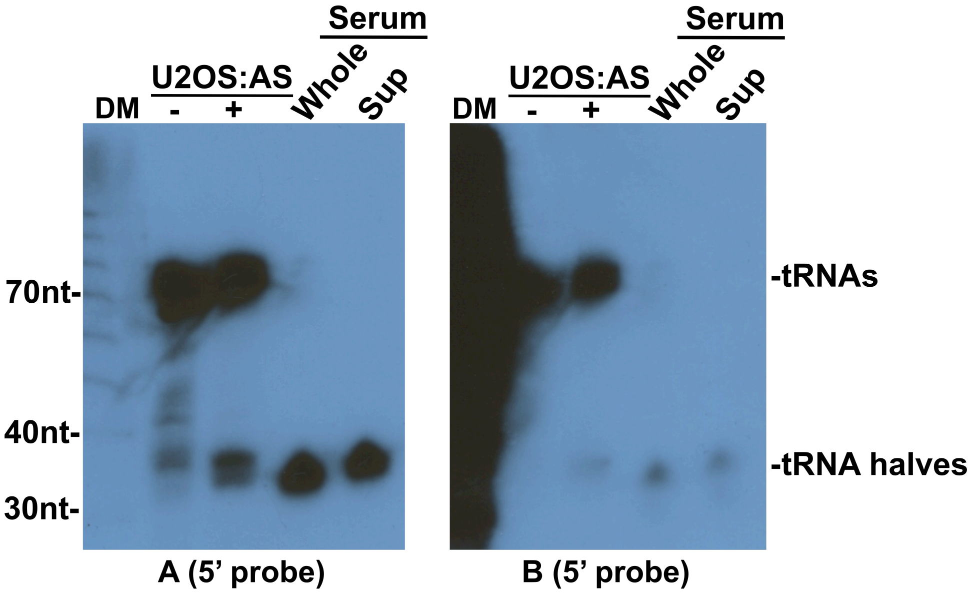

Figure S2 – Scarcity of 5' tRNA-Asn halves in mouse serum. Northern blot analysis of RNA extracted from U2OS cells cultured in the absence (-) or presence (+) of sodium arsenite (AS), or from 0.4 ml of mouse serum, or from the supernatant (Sup) after ultracentrifugation of 0.4 ml of mouse serum at 110000g. The blot was hybridized to a ^{32}P-end-labeled oligonucleotide probe complementary to the 5' end of tRNA-Gly-GCC (A) or the 5' end of tRNA-Asn-GTT (B). The blot hybridized to the 5' end of tRNA-Gly-GCC was exposed to an X-ray film for 25 minutes, while the blot hybridized to the 5' end of tRNA-Asn was exposed for 5 days. The positions of full length tRNAs and tRNA halves are indicated on the right. DM: decade markers.
